# LRH-1 expression patterns in breast cancer tissues are associated with tumour aggressiveness

**DOI:** 10.18632/oncotarget.18886

**Published:** 2017-07-28

**Authors:** Jia-Min B. Pang, Ramyar Molania, Ashwini Chand, Kevin Knower, Elena A. Takano, David J. Byrne, Thomas Mikeska, Ewan K.A. Millar, Cheok Soon Lee, Sandra A. O’Toole, Colin Clyne, Kylie L. Gorringe, Alexander Dobrovic, Stephen B. Fox

**Affiliations:** ^1^ Department of Pathology, Peter MacCallum Cancer Centre, Victorian Comprehensive Cancer Centre, Melbourne, VIC 3000, Australia; ^2^ Translational Genomics & Epigenomics Laboratory, Olivia Newton-John Cancer Research Institute, Heidelberg, VIC 3084, Australia; ^3^ Department of Medicine, University of Melbourne, Parkville, VIC 3010, Australia; ^4^ Cancer Drug Discovery Laboratory, Hudson Institute of Medical Research, Clayton, VIC 3168, Australia; ^5^ School of Cancer Medicine, La Trobe University, Bundoora, VIC 3084, Australia; ^6^ Department of Pathology, University of Melbourne, Parkville, VIC 3010, Australia; ^7^ The Kinghorn Cancer Centre and Garvan Institute of Medical Research, Darlinghurst, NSW 2010, Australia; ^8^ Department of Anatomical Pathology, South Eastern Area Pathology Service, St George Hospital, Kogarah, NSW 2217, Australia; ^9^ School of Medical Sciences, University of New South Wales, Kensington, NSW 2052, Australia; ^10^ Discipline of Pathology, School of Medicine, University of Western Sydney, Campbelltown, NSW 2751, Australia; ^11^ Department of Tissue Pathology and Diagnostic Oncology, Royal Prince Alfred Hospital, Camperdown, NSW 2005, Australia; ^12^ Cancer Pathology, Bosch Institute, University of Sydney, NSW 2006, Australia; ^13^ Sydney Medical School, University of Sydney, NSW 2006, Australia; ^14^ Cancer Genomics Program, Peter MacCallum Cancer Centre, Melbourne, VIC 3000, Australia; ^15^ Sir Peter MacCallum Department of Oncology, University of Melbourne, Parkville, VIC 3010, Australia

**Keywords:** immunohistochemistry, NR5A2, DNA methylation, breast carcinoma, estrogen signalling

## Abstract

The significance and regulation of liver receptor homologue 1 (LRH-1, *NR5A2*), a tumour-promoting transcription factor in breast cancer cell lines, is unknown in clinical breast cancers. This study aims to determine LRH-1/*NR5A2* expression in breast cancers and relationship with DNA methylation and tumour characteristics. In The Cancer Genome Atlas breast cancer cohort *NR5A2* expression was positively associated with intragenic CpG island methylation (1.4-fold expression for fully methylated versus not fully methylated, p=0.01) and inversely associated with promoter CpG island methylation (0.6-fold expression for fully methylated versus not fully methylated, p=0.036). LRH-1 immunohistochemistry of 329 invasive carcinomas and ductal carcinoma *in situ* (DCIS) was performed. Densely punctate/coarsely granular nuclear reactivity was significantly associated with high tumour grade (p<0.005, p=0.033 in invasive carcinomas and DCIS respectively), negative estrogen receptor status (p=0.008, p=0.038 in overall cohort and invasive carcinomas, respectively), negative progesterone receptor status (p=0.003, p=0.013 in overall cohort and invasive carcinomas, respectively), *HER2* amplification (overall cohort p=0.034) and non-luminal intrinsic subtype (p=0.018, p=0.038 in overall cohort and invasive carcinomas, respectively). These significant associations of LRH-1 protein expression with tumour phenotype suggest that LRH-1 is an important indicator of tumour biology in breast cancers and may be useful in risk stratification.

## INTRODUCTION

Liver receptor homologue 1 (LRH-1, *NR5A2*) is a nuclear receptor and acts as a transcription factor with roles in embryogenesis, steroid and cholesterol metabolism, inflammation, and in several cancers, including gastrointestinal malignancies and breast cancer [1–9]. In breast cancer cell lines, multiple studies have shown that LRH-1 has tumour-promoting roles in estrogen production, estrogen receptor (ER) signalling, cell cycle control, and cellular migration and invasion [1, 7, 8, 10–15]. LRH-1 also stimulates local oestrogen production by upregulating aromatase activity in breast adipose tissue [10, 12, 13] and by enhancing the effect of prostaglandin E_2_ on aromatase expression [12]. In addition, LRH-1 and ER each promote the expression of the other [11, 15], share many binding sites [14], and co-operatively regulate the expression of ER target genes [11, 14].

LRH-1 shows ER- independent actions too, such as modulating expression of genes involved in cell cycle control including upregulating the expression of *CCND1* [7], *MYC* [7] and *BCL2* [7], and suppressing *CDKN1A* expression [1]. Reduction of cell proliferation upon LRH-1 knockdown occurs in a p53-independent manner [1] and results in an increased proportion of cells in the G_0_/G_1_ phase of the cell cycle and reduction of cells in the S and G_2_/M phases [15]. Compared with ER-positive breast cancer cells, the anti-proliferative effect of LRH-1 knockdown on the cell cycle is more pronounced in MCF-7 in the absence of E_2_ [15], in MCF7- derived anti-estrogen-resistant cell lines (MCF7/LCC2 and MCF7/LCC9) and in the ER-negative cell line BT-549 [1, 7]. This suggests that LRH-1 may have a greater role in driving cell proliferation in breast cancer cells in the absence of functional ER, perhaps by providing an alternative mechanism for regulation of ER target genes. Indeed, higher LRH-1 expression is present in MCF7/LCC2 and MCF7/LCC9 cell lines compared with parental MCF7 cells [7], and overexpression of LRH-1 in the ER-negative cell line MDA-MB-231 results in significant up-regulation of the ER target gene *GREB1* [11].

Although LRH-1 mediates processes that promote tumorigenesis in both estrogen-driven and estrogen-independent breast cancer cells, the direct role of LRH-1 in human breast cancer remains unexplored. Most LRH-1 studies have been performed on breast cancer cell lines and data from breast cancer tissues is limited, both regarding LRH-1 expression and the relationship of LRH-1 with tumour biology. Moreover, although LRH-1 expression is influenced by ER in ER-positive breast cancer, very little is known about alternative mechanisms controlling LRH-1 expression, in particular how it is regulated in ER-negative breast tumours, and in ER-positive breast cancers resistant to anti-estrogenic therapy.

**Figure 1:**
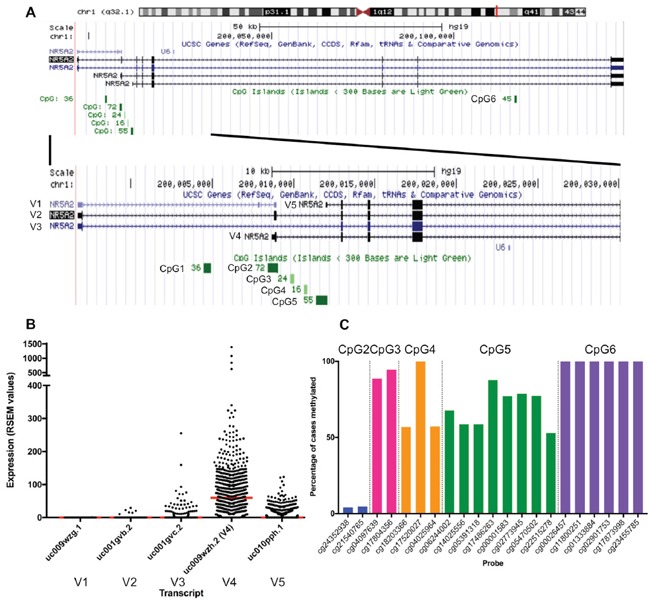
**(A)** Genomic region of NR5A2 showing the five transcripts (V1-V5) and CpG islands (CpG1 – CpG6). **(B)** mRNA expression of *NR5A2* transcripts in TCGA invasive breast cancer cohort. RSEM, RNA-Seq by Expectation Maximization. Red line indicates median. **(C)** Methylation frequencies of HM450 probes within *NR5A2* CpG islands CpG2-CpG6 in TCGA invasive breast cancer cohort.

LRH-1 is encoded by the *NR5A2* gene which is located on chromosome 1 at band q32.1. There are at least five described *LRH-1* mRNA transcripts [2, 16–20], generated by different transcription initiation sites as well as alternative splicing, four of which are associated with protein products [21] (Table 1, Figure 1). Regulation of these transcripts may be controlled by methylation, as six CpG islands are present in the gene region. A 501 amino acid protein, first described and named variant 4 by Thiruchelvam *et al*. in 2011 [20], is reported to be the predominant mRNA variant in breast cancer cell lines and to be highly estrogen regulated compared with other variants [20]. Therefore the aims of this study are to assess the importance of LRH-1 *in situ* and invasive breast cancer, in particular to investigate (1) *NR5A2* transcript expression in invasive breast cancers (2) the role of DNA methylation in regulating the expression of *NR5A2*, (3) the level and pattern of expression of LRH-1 protein in a cohort of ductal carcinoma *in situ* (DCIS) and invasive breast carcinomas to assess its potential role in tumour progression and (4) the relationship between LRH-1 expression and clinicopathological features.

**Table 1:** Baseline characteristics of women in this study by colposcopy compliance

A. *NR5A2* transcripts				
Genomic sequence (GRCh37/hg19)	Number of exons	RefSeq accession	RNA accession	Protein length
chr1:199,996,730-200,008,923	3	NM_205860	AF124248.1	No protein
chr1:199,996,730-200,146,550	8	NM_205860	NM_205860.2	541aa
chr1:199,996,730-200,146,550	7	NM_003822	NM_003822.4	495aa
chr1:200,008,658-200,146,550	7	NM_001276464 (“variant 4”)	AK304344.1 (“variant 4”)	501aa
chr1:200,011,953-200,146,550	6	NM_001276464	NM_001276464.1	469aa

B. CpG islands within *NR5A2*				
CpG island	Genomic location [16, 22] (GRCh37/hg19)	Number of CpG dinucleotides
1	chr1:200,004,475-200,004,933	36
2	chr1:200,008,393-200,009,047	72
3	chr1:200,009,808-200,010,036	24
4	chr1:200,010,626-200,010,832	16
5	chr1:200,011,401-200,012,055	55
6	chr1:200,116,697-200,117,204	45

**Figure 2:**
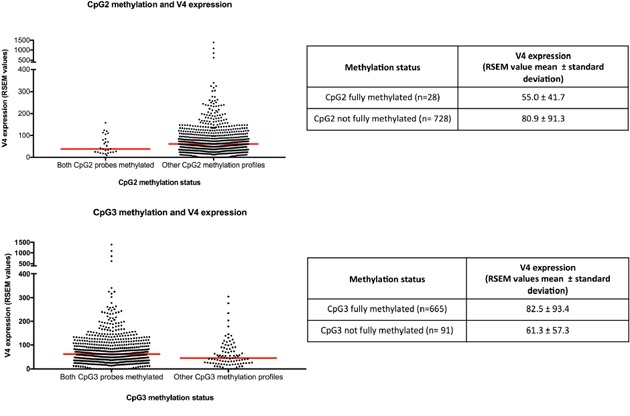
CpG2 and CpG3 methylation and *NR5A2* variant 4 expression RSEM, RNA-Seq by Expectation Maximization. Red line indicates median.

## RESULTS

### *NR5A2* mRNA expression and its relationship to DNA methylation using The Cancer Genome Atlas (TCGA) data

Six CpG islands are located within the *NR5A2* gene [16, 22] (Table 1, Figure 1A) with several CpG islands associated with variant 4. The second CpG island (CpG2) of these six is situated immediately upstream of variant 4 and four intragenic CpG islands (third, fourth, fifth and sixth CpG islands - CpG3, CpG4, CpG5 and CpG6, respectively) are located nearby, suggesting that methylation of these islands may have a role in regulating expression of variant 4. The first CpG island (CpG1) is located approximately 4 kb upstream of variant 4.

Isoform-specific expression data and methylation data was available for 756 samples. ER, PR, and HER2 status were available for 371, 333, and 468 cases, respectively. The majority of cases were ER positive (86.8%, 322/371), PR positive (70.6%, 235/333), and HER2 negative (80.8%, 378/468). No information regarding tumour grade was available.

Similar to published breast cancer cell line data [15, 20, 23], variant 4 was the predominantly expressed transcript in invasive breast cancers in the TCGA cohort (Figure 1). Variant 4 expression levels were significantly higher in ER-positive tumours compared with ER-negative tumours (ER positive mean 89.6 ± 116.6 RNA-Seq by Expectation Maximization (RSEM) values; ER negative mean 65.7 ± 64.9 RSEM values, p=0.011). This is not a consequence of the frequent copy number gain of *NR5A2* in ER-positive tumours, as copy number and mRNA expression are not positively correlated in TCGA data (Pearson r=-0.093). Variant 4 expression did not differ significantly based on PR or HER2 status (p=0.101 and p=0.079 respectively).

CpG6 was universally methylated at all six probe sites. CpG2 was the least frequently methylated, with just 4.9% (40/756) of cases methylated for both CpG2 probes. CpG3, CpG4, and CpG5 showed some degree of methylation in the vast majority of cases (methylation present in 95.2% (720/756), 100%, and 95.8% (724/756) of cases, respectively), however methylation was somewhat heterogeneous (all probes within CpG island methylated in 88.0% (665/756), 48.1% (364/756), 31.6% (239/756) of cases, respectively) (Figure 1).

The expression of variant 4 was significantly lower in the 28 cases methylated at both CpG2 probes compared with cases not showing this methylation profile (p=0.036, Figure 2). In contrast, cases methylated at both CpG3 probes (n= 665) showed higher variant 4 expression compared with cases unmethylated at one or both probes (p=0.01, Figure 2). All cases methylated at both CpG2 probes were also methylated at both CpG3 probes, while 95.8% (637/665) of cases methylated at both CpG3 sites were not fully methylated at CpG2. The methylation status of CpG4 and CpG5 were not significantly associated with variant 4 expression.

### LRH-1 protein expression

**Figure 3:**
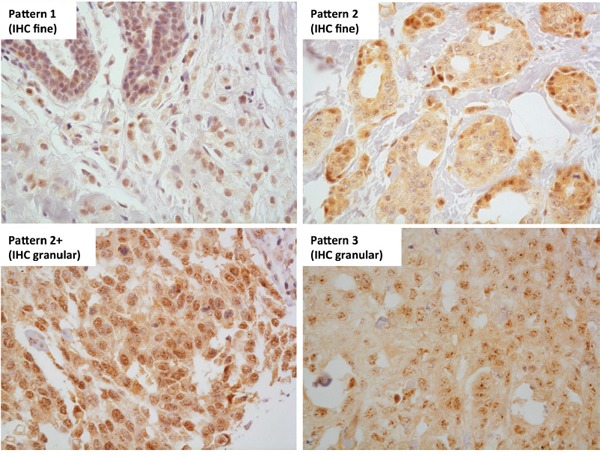
LRH-1 IHC nuclear reactivity patterns Four patterns of LRH-1 IHC nuclear reactivity were observed - a finely granular pattern, similar to that seen in normal breast epithelium (pattern 1), a sparse punctate pattern (pattern 2), a dense punctate pattern (pattern 2+), and a coarse granular pattern (pattern 3). Patterns 1 and 2 were classified as LRH-1 IHC fine and patterns 2+ and 3 were classified as LRH-1 IHC granular. 400x magnification.

While *NR5A2* mRNA was associated with ER status in invasive breast cancer, post-translational regulation of LRH-1 protein may affect such associations with breast cancer characteristics. Thus we evaluated LRH-1 protein using immunohistochemistry (IHC), successfully obtaining results from 329 cases of breast cancer, comprising 175 DCIS (146 pure DCIS and 29 DCIS occurring synchronously with invasive carcinoma) and 154 invasive carcinomas. When present, IHC nuclear reactivity was observed in all epithelial cells throughout the tumours, with four patterns of nuclear positivity observed (Figure 3). Nuclear positivity showed a finely dispersed pattern (pattern 1), a sparse punctate pattern (pattern 2), a dense punctate pattern (pattern 2+), and a coarse granular pattern (pattern 3). Patterns 2, 2+, and 3 also showed finely dispersed nuclear positivity in the background. Most cases showed uniformity of nuclear pattern; where this was not the case the dominant pattern was recorded. 1.2% of cases (4/329) showed no nuclear positivity. The frequencies of patterns 1, 2, 2+, and 3 staining were 28.3% (93/329), 50.5% (166/329), 14.0% (46/329), and 6.1% (20/329), respectively. Cases with no IHC staining or pattern 1 or 2 staining were considered to be ‘IHC fine’. Patterns 2+ and 3 were considered to be ‘IHC granular’.

There was no difference in LRH-1 staining between DCIS and invasive carcinoma (p=0.411, Table 2). In the overall cohort (Table 2), the IHC-granular group was significantly associated with high grade (p<0.0005), ER negativity (p=0.008), PR negativity (p=0.003), *HER2* amplification (p=0.034) and non-luminal intrinsic subtypes (p=0.018) compared with cases not showing this pattern. There was a trend for LRH-1 granular tumours to be larger but this was not statistically significant (p=0.054). There was no association with age or lymph node status. When separating out by tumour type, invasive tumours showing IHC granular staining were significantly correlated with high grade (p<0.0005), ER negativity (p=0.038), PR negativity (p=0.013), and non-luminal intrinsic subtypes (p=0.038) (Table 2). In DCIS, IHC-granular staining patterns were associated with high nuclear grade (p=0.033) (Table 2).

**Table 2:** LRH-1 IHC and tumour phenotype

	Overall cohort (n=329)			Invasive carcinoma (n=154)			DCIS (n=175)		
	IHC granular	IHC fine	p-value	IHC granular	IHC fine	p-value	IHC granular	IHC fine	p-value
**Number**	66	263		34	120		32	143	
**Tumour type**									
Invasive	34	120	0.411	N/A	N/A	N/A	N/A	N/A	N/A
DCIS	32	143							
**DCIS type**									
Pure	N/A	N/A	N/A	N/A	N/A	N/A	29	117	0.298
Mixed							3	26	
nd							0	0	
**Grade**									
High/ grade 3	45	111	**<0.0005**	30	81	**<0.0005**	15	30	**0.033**
Non-high/ grade 1 or 2	5	69		1	38		4	31	
nd	16	83		3	1		13	82	
**Size**									
**≤20 mm**	16	87	0.054	12	65	0.113	4	22	0.392
>20 mm	33	93		20	55		13	38	
**Age (years)**									
**<50**	9	54	0.105	5	36	0.120	4	18	0.757
**≥50**	39	119		28	85		11	34	
**Lymph node status**									
Positive	N/A	N/A	N/A	8	23	0.777	N/A	N/A	N/A
Negative				8	29				
**ER status**									
Positive	24	142	**0.008**	6	45	**0.038**	18	97	0.138
Negative	41	113		28	75		13	38	
nd	1	8		0	0		1	8	
**PR status**									
Positive	16	117	**0.003**	3	36	**0.013**	13	81	0.153
Negative	48	138		31	83		17	55	
nd	2	2		0	1		2	7	
***HER2*** **amplification**									
Amplified	17	37	**0.039**	7	14	0.255	10	23	0.077
Non-amplified	48	220		27	106		21	114	
nd	1	6		0	0		1	6	

**nd** no data available, **N/A** non-applicable.

## DISCUSSION

*In vitro* studies have shown important regulatory functions for LRH-1 in breast cancer cell lines [1, 7, 8, 11, 13, 14]. However, little is known about LRH-1 in clinical breast cancer samples or its role in the biology of breast cancer, its relationship to *in situ* to invasive transition, how this might be regulated or its association with tumour behaviour. In this study, LRH-1 expression in breast cancer was explored, including relationships between methylation and gene expression; between gene expression, HER2 and hormonal status; and between protein expression and breast cancer characteristics.

To explore the role of methylation in the regulation of the predominantly expressed *NR5A2* transcript, variant 4, we identified the presence of CpG islands within the *NR5A2* gene, including in the presumed promoter region and the gene body of variant 4 itself. The presence of these CpG islands suggested that DNA methylation may have an important role in regulating *NR5A2* gene expression. In keeping with this theory, analysis of TCGA Infinium HumanMethylation450 array data revealed that methylation of CpG2 was associated with lower levels of *NR5A2* variant 4 expression, whereas methylation of CpG3 was associated with higher *NR5A2* variant 4 expression. Although this appears paradoxical, this observation is consistent with the evidence that CpG-island methylation is context-dependent, whereby methylation of CpG-island promoter sites is associated with transcriptional silencing while gene body CpG-island methylation is not associated with gene silencing and may instead be associated with transcription [24].

The expression of *NR5A2* variant 4 has been reported in both ER-positive and ER-negative breast cancer cell lines [20] and our analysis of mRNA sequencing data of TCGA invasive breast cancer cohort found that variant 4 was also the predominantly expressed *NR5A2* transcript in primary breast cancers. Expression of this variant was higher in ER-positive breast tumours compared with ER-negative tumours, supporting the findings of Muscat *et al*. [25], who in their study of 66 invasive breast cancers and 50 normal breast samples observed that *NR5A2* mRNA expression was greater in ER-positive tumours compared with ER-negative tumours and was negatively correlated with tumour grade [25]. High levels of *NR5A2* mRNA in ER-positive tumours may be related to the role of LRH-1 in the ER transcriptional program, but high levels of *NR5A2* mRNA do not necessarily lead to high levels of LRH-1 protein or LRH-1 activity. Variant 4 is subject to E_2_-mediated degradation, resulting in a significantly shorter half-life in ER-positive cells compared with ER-negative cells [23]. In addition, the resultant protein has also been reported to be more stable in ER-negative breast cancer cells compared with ER-positive cells [23]. These findings support our observation of increased IHC reactivity (IHC granular pattern) in ER-negative carcinomas compared with ER-positive tumours. Furthermore, expression of *NR5A2* mRNA in ER-negative tumours is strongly correlated with expression of multiple co-regulators and upregulation of ER-related genes, relationships which are not observed in ER-positive tumours [9]. Taken together, the data suggest that there is differential modulation of LRH-1 activity between ER-positive and ER-negative tumours, and that LRH-1 has roles in ER-associated, but ER-independent pathways in ER-negative tumours, similar to the observations made in breast cancer cell lines [11].

Our LRH-1 IHC data support the ER-positive/ER-negative differential. Tumours with predominantly coarse staining were more likely to be ER-negative and also display aggressive phenotypic features such as being high grade. This result is in contrast to the two previous studies examining LRH-1 expression by IHC in breast cancers that reported LRH-1 expression to be associated with favourable tumour characteristics [15, 26]. The discordant findings are likely to be due to the different IHC primary antibodies and scoring criteria used. Both the previous studies (Annicotte *et al*. [15] and Miki *et al*. [26]) used an anti-LRH-1 IHC antibody directed only towards the 541 amino acid protein resulting from the mRNA transcript NM_205860 (uc001gvb.2). This transcript was observed in TCGA breast cancer cohort to be expressed at very low levels (median 0 RSEM value, range 0-255) compared with the predominant transcript, variant 4 (median 59.9 RSEM value, range 0-1390) and therefore may not truly reflect total LRH-1 expression in breast cancers. In contrast, the antibody used in this study recognizes all forms of the LRH-1 protein. As for the scoring methodology, the IHC scoring criteria used by Annicotte *et al*. were not stated [15], while Miki *et al*. quantified only the percentage of nuclear reactivity, considering tumours with staining in at least 10% of tumour nuclei to be positive for LRH-1 expression [26].

LRH-1 is subject to various types of post-translational modifications including phosphorylation, acetylation, ubiquitination and SUMOylation [27]. SUMOylation refers to the covalent attachment of the small ubiquitin-related modifier (SUMO) to specific lysine residues [28]. SUMOylation of LRH-1 leads to sequestration of LRH-1 in promyelocytic leukaemia (PML) protein nuclear bodies, localising as discrete nuclear dots, in contrast to unSUMOylated LRH-1, which is distributed diffusely in the nucleus [29]. Sequestrated LRH-1 in PML protein nuclear bodies is transcriptionally inactive and is thought to serve as a LRH-1 reservoir [29]. Low intra-nuclear concentrations of LRH-1 lead to de-SUMOylation and release of LRH-1 from the PML protein nuclear bodies and an associated change in nuclear distribution of LRH-1 from discrete nuclear dots to a diffuse nuclear distribution [29]. Therefore the coarse granular IHC staining pattern could represent breast tumours with stored SUMOylated intra-nuclear LRH-1 protein in PML protein nuclear bodies due to high overall LRH-1 levels. The observation of coarse granular IHC staining occurring on a background of diffuse nuclear staining is consistent with this hypothesis (Figure 3).

If coarsely granular nuclear LRH-1 IHC staining is indeed indicative of tumours with higher intra-nuclear levels of LRH-1, the association of this staining pattern with aggressive phenotypic features in breast cancer is consistent with previously reported associations of LRH-1 with tumorigenic functions in breast cancer cells [1, 7, 8, 11, 14], the association of LRH-1 knockdown with down-regulation of genes that are overexpressed in high-grade tumours and associated with poor outcome [7], and with the observation that high *NR5A2* mRNA expression in conjunction with low *CDKN1A* expression conferred poor disease-free survival in breast cancer patients in TCGA dataset [1].

In conclusion, the nuclear receptor LRH-1 has previously been reported to have tumorigenic functions in breast cancer cell lines, raising the possibility that it may have similar roles in clinical breast cancer. Analysis of TCGA cohort of invasive breast carcinomas revealed that, similar to breast cancer cell lines, variant 4 was the predominantly expressed transcript and expression of variant 4 was associated with DNA methylation status. Distinct IHC nuclear reactivity patterns were identified for LRH-1 ranging from finely dispersed to coarsely granular staining, with densely punctate and coarsely granular nuclear staining being associated with aggressive breast cancer characteristics, suggesting that LRH-1 expression is informative of breast cancer biology. This may be of clinical relevance as IHC can provide a relatively straightforward method of identifying cases potentially at higher risk of poor clinical outcome and therefore can be used to risk stratify patients for appropriate treatment selection and may be indicative of response to proposed LRH-1 antagonists [23, 30–33].

## MATERIALS AND METHODS

### *NR5A2* mRNA expression and DNA methylation

The Cancer Genome Atlas (TCGA) breast invasive carcinoma cohort was utilized to obtain mRNA expression and DNA methylation data in a large number of breast cancer cases. Processed gene isoforms expression data (RNA-Seq version 2, level 3 data, accessed 28 January 2016), HM450 array data (level 3 data, accessed 28 January 2016), and clinical and histopathological data from invasive breast cancer cohort (breast invasive carcinoma) [34] were obtained from the TCGA data portal.

Gene isoforms expression values and methylation level of HM450 were reported as normalized RSEM (RNA-Seq by Expectation Maximization) count [35] and beta value, respectively. Quality control and batch effects analysis were performed using Relative Loge Expression plot and unsupervised method including principal component analysis (PCA).

The RNA sequencing data was examined for expression of isoforms uc009wzg.1, uc001gvb.2, uc001gvc.2, uc009wzh.2, and uc010pph.1, corresponding to the five *NR5A2* transcripts, variant 4 being uc009wzh.2.

The HM450 methylation array data was examined for methylation of the CpG island associated with the presumed promoter region of variant 4 (CpG2) and of the four intragenic CpG islands (CpG3, CpG4, CpG5, CpG6), specifically methylation of two CpG2 probes (cg24352938, cg21540765), two CpG3 probes (cg04097639, cg17804356), three CpG4 probes (cg18203366, cg17520027, cg04025964), eight CpG5 probes (cg06244002, cg14025556, cg05391318, cg17486263, cg00001583, cg02773945, cg 05470502, cg22515278) and six CpG6 probes (cg00026457, cg11800251, cg01333884, cg02901753, cg17873998, cg23455785). For each probe, samples with a beta value of ≥0.2 were regarded as methylated.

TCGA cases were considered positive for ER and PR if there was reported staining in at least 10% of tumour cells. The exact percentage of positive tumour nuclei was not stated for cases with <10% reactivity, precluding the use of a 1% cut-off to define hormone receptor positivity. For HER2, cases with equivocal immunohistochemical staining were not included in the analysis.

### Patient cohorts

Invasive carcinomas with associated DCIS were obtained from the Peter MacCallum Cancer Centre pathology department from archived diagnostic cases between 2000 and 2011. The DCIS cases were obtained from archived diagnostic cases from the pathology departments of Peter MacCallum Cancer Centre between 2004 and 2013, and Royal Prince Alfred Hospital between 1992 and 2004. Haematoxylin and eosin-stained sections of all cases were reviewed by a pathologist to confirm diagnosis and the tumour grade of invasive breast carcinomas was determined using modified Bloom and Richardson with grade 3 tumours considered to be high grade. DCIS cases were reviewed and nuclear grade was classified as low, intermediate, and high according to consensus guidelines [36].

### LRH-1 and biomarker protein expression

LRH-1 protein expression was assessed by immunohistochemistry on 3 μm-thick sections of formalin-fixed, paraffin-embedded tissue microarrays containing DCIS and invasive breast cancer cases. Sections were de-waxed and hydrated through ethanol to water. Antigen retrieval was performed using High pH EnVision FLEX Target Retrieval Solution (Dako) at 124°C at 15-16 PSI for 4 minutes. Sections were incubated with the anti-LRH-1 rabbit polyclonal antibody (Catalog number HPA005455, Sigma Aldrich, St. Louis, MO) at 1:100 dilution overnight at 4°C, followed by detection using the EnVision FLEX/HRP DAB detection kit (Dako).

The LRH-1 antibody used is directed towards a 147 amino acid sequence present in all versions of the LRH-1 protein and its specificity was assessed by Western blot (Supplementary Figure 1). This antibody has been documented by the Human Protein Atlas database to stain epithelial cells of the majority of breast cancers, where ubiquitous nuclear staining with a speckled pattern is expected [37, 38]. ER, progesterone receptor, CK5/6, EGFR and Ki-67 immunohistochemistry and *HER2* silver-enhanced *in situ* hybridization were performed on the LRH-1 IHC cohort using tissue microarray sections, as previously described [39, 40]. For ER and PR, tumours were regarded as positive if at least 10% of tumour nuclei were reactive, regardless of intensity. A cut-off of 10% tumour cell positivity was chosen to account for non-specific reactivity. *HER2* amplification status was assessed by counting signals in twenty tumour nuclei, where possible. Tumours were considered *HER2*-amplified if the average number of signals per tumour nucleus was at least 6 [41].

Tumours were classified into intrinsic subtypes according to the St Gallen International Expert Consensus 2013 [42]. Luminal A-like and luminal B-like tumours were considered luminal tumours, while HER2 positive tumours without ER or PR positivity and triple negative (including basal-like) tumours were considered non-luminal tumours.

Approval for the project was obtained from the ethics committees of Peter MacCallum Cancer Centre (project numbers 02/26, 10/16, and 00/81) and Royal Prince Alfred Hospital (project HREC/11/RPAH/126).

### Statistical analysis

Comparisons of continuous data between two groups was evaluated by Mann-Whitney U test. Fisher's exact probability test was used to assess 2×2 contingency tables. For each comparison, a two-tailed p value of 0.05 or less was considered to be statistically significant. All statistical analyses were performed using IBM SPSS version 22.0 (IBM Corporation, NY).

## SUPPLEMENTARY MATERIALS FIGURES



## Abbreviations

DCIS: Ductal carcinoma *in situ*
ER: Estrogen receptor
IHC: Immunohistochemistry
HM450: Infinium HumanMethylation450 array
LRH-1: Liver receptor homologue 1
PML: Promyelocytic leukaemia
PR: Progesterone receptor
RSEM: RNA-Seq by Expectation Maximization
TCGA: The Cancer Genome Atlas
SUMO: Small ubiquitin-related modifier
