# Supplementary material for: LRH-1 expression patterns in breast cancer tissues are associated with tumour aggressiveness
Source: Oncotarget. 2017 Jul 28;8(48):83626–36. doi: 10.18632/oncotarget.18886 (PMC5663541; doi:10.18632/oncotarget.18886)
Supplement: Supplementary file 1 [file oncotarget-08-83626-s001.pdf]

LRH-1 expression patterns in breast cancer tissues are associated with tumour aggressiveness

SUPPLEMENTARY MATERIALS

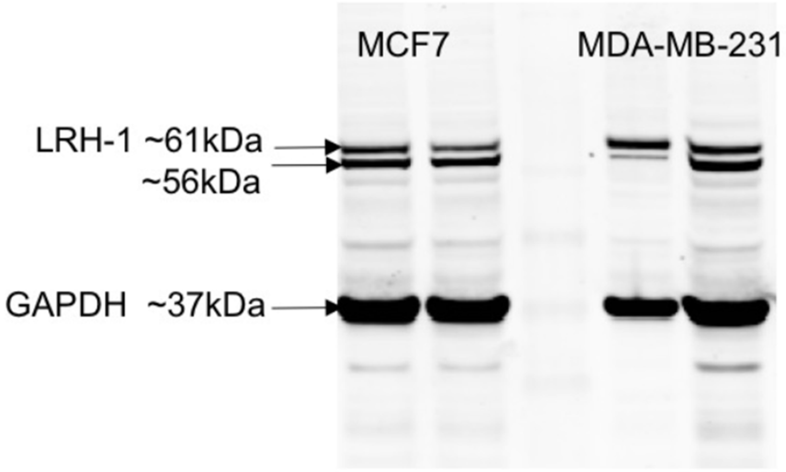

Supplementary Figure 1: Western blot using LRH-1 antibody identifies two isoforms in the ERα positive breast cancer cell line MCF7 and basal-like ERα negative breast cancer cell line, MDA-231. GAPDH is included as a loading control.
